# 

**Published:** 1861-08

**Authors:** 


Vol. II. AUGUST, 1861. No. 8.

### THE CHICAGO MEDICAL EXAMINER

A MONTHLY JOURNAL, DEVOTED TO THE

Educational, Scientific [illegible] Practical Interests,

[illegible]

MEDICAL PROFESSION.

EDITED BY

N. S. DAVIS, M. D.,

PROFESSOR OF PRINCIPLES AND PRACTICE OF MEDICINE AND OF CLINICAL MEDICINE, IN THE MEDICAL DEPARTMENT OF LIND UNIVERSITY, ETC., ETC.

AND

FRANK W. REILLY, M. D.

TERMS, $2.00 PER ANNUM, IN ADVANCE.

CHICAGO:

TRIBUNE BOOK AND JOB PRINT, No. 51 CLARK STREET.

